# A New Bloody Pulp Selection of Myrobalan (*Prunus cerasifera* L.): Pomological Traits, Chemical Composition, and Nutraceutical Properties

**DOI:** 10.3390/foods12051107

**Published:** 2023-03-05

**Authors:** Francesco Sottile, Assunta Napolitano, Natale Badalamenti, Maurizio Bruno, Rosa Tundis, Monica Rosa Loizzo, Sonia Piacente

**Affiliations:** 1 Department of Architecture, University of Palermo, Viale delle Scienze, 90128 Palermo, PA, Italy; 2 Interdepartmental Research Center “Bio-Based Reuse of Waste from Agri-Food Matrices” (RIVIVE), University of Palermo, Viale delle Scienze, 90128 Palermo, PA, Italy; 3 Department of Pharmacy, University of Salerno, 84084 Fisciano, SA, Italy; 4 Department of Biological, Chemical and Pharmaceutical Sciences and Technologies, University of Palermo, Viale delle Scienze, Parco d’Orleans II, 90128 Palermo, PA, Italy; 5 NBFC—National Biodiversity Future Center, 90133 Palermo, PA, Italy; 6 Department of Pharmacy, Health and Nutritional Sciences, University of Calabria, 87036 Rende, CS, Italy

**Keywords:** *Prunus cerasifera*, myrobalan, LC-MS analysis, antioxidant activity, lipase and carbohydrate hydrolyzing enzymes inhibitory effects

## Abstract

A new accession of myrobalan (*Prunus cerasifera* L.) from Sicily (Italy) was studied for the first time for its chemical and nutraceutical properties. A description of the main morphological and pomological traits was created as a tool for characterization for consumers. For this purpose, three different extracts of fresh myrobalan fruits were subjected to different analyses, including the evaluation of total phenol (TPC), flavonoid (TFC), and anthocyanin (TAC) contents. The extracts exhibited a TPC in the range 34.52–97.63 mg gallic acid equivalent (GAE)/100 g fresh weight (FW), a TFC of 0.23–0.96 mg quercetin equivalent (QE)/100 g FW, and a TAC of 20.24–55.33 cyanidine-3-*O*-glucoside/100 g FW. LC-HRMS analysis evidenced that the compounds mainly belong to the flavonols, flavan-3-ols, proanthocyanidins, anthocyanins, hydroxycinnamic acid derivatives, and organic acids classes. A multitarget approach was used to assess the antioxidant properties by using FRAP, ABTS, DPPH, and *β*-carotene bleaching tests. Moreover, the myrobalan fruit extracts were tested as inhibitors of the key enzymes related to obesity and metabolic syndrome (α-glucosidase, α-amylase, and lipase). All extracts exhibited an ABTS radical scavenging activity that was higher than the positive control BHT (IC_50_ value in the range 1.19–2.97 μg/mL). Moreover, all extracts showed iron-reducing activity, with a potency similar to that of BHT (53.01–64.90 *vs* 3.26 μM Fe(II)/g). The PF extract exhibited a promising lipase inhibitory effect (IC_50_ value of 29.61 μg/mL).

## 1. Introduction

Among fresh fruit species, plums (genus *Prunus*) play a non-determinant role in terms of production and cultivated surfaces, yet it is traditionally found in many areas characterized by temperate climates. It is usually considered a minor stone fruit together with apricot mostly because it is compared to peach and nectarines, which accounts for wider diffusion and growing areas [1]. The species has a complex botanical classification because several species are traced back to the name plum, and its hybridizations, natural and/or induced, are widespread [2]. Domestic or European plums (*Prunus domestica* L.) are generally used for processing into dried plums, while Japanese plums (*Prunus salicina* Lindl.) are almost exclusively used for fresh consumption. The names of the two species highlight the links between the origin and geographical distribution, with the former being more closely associated with the old continent and the latter with the Asian continent. However, these are genetically distant species characterized by different ploidy levels.

Myrobalan (*Prunus cerasifera* L.) is a diploid, widespread species in the Mediterranean. This species is credited with the origin of European plums via hybridization with the tetraploid *P. Spinosa*, and for this reason, myrobalan is considered genetically close to *P. domestica*, although with different ploidy [1]. It is widely used as rootstock for both plum and apricot trees, more rarely for peach [3,4] thanks mainly to its ability to produce adventitious roots that facilitate its propagation [5,6].

In many traditional fruit-growing areas, where intensive agriculture has not taken over, there are several edible fruiting myrobalan accessions, often small (hence referred to as cherry-plum), selected by farmers and passed down because of the consumption related to the gastronomic traditions of indigenous peoples [7]. For this reason, many studies have focused on characterizing the accessions that are locally grown and highly valued by consumers, often for the taste qualities of the fruits as well as their early ripening time [8]. This approach is very often based on the analysis of morphological data through the adoption of specific descriptor lists studied and approved on a global scale [9]. The morphological traits of flowers, leaves, fruits, and seeds are studied, as well as wood, crown, bud characteristics, tree habit, and phenotypic behavior by the age of maturity and flowering. More recently, it has been proposed to combine these observations with a molecular-scale evaluation model, which, however, becomes effective only when morphological analysis fails to separate different accessions and, in any case, only in the presence of an adequate bank of genetic information [10]. Over the past decade, on an international scale, many research centers have initiated several programs for biodiversity conservation and characterization in accordance with the provisions of the International Treaty on Plant Genetic Resources for Agriculture and Food [11]. This important agreement has given all participating countries a responsibility in the conservation of indigenous genetic resources through the development of national plans in which the main objective is to enable a description of biodiversity with comparable and recognized patterns. Many innovative approaches have been developed for preserving plum biodiversity from the risk of erosion and/or extinction [12]. It is well known that the myrobalan fruits are usually rich in fibers and antioxidants, evidencing an important role in terms of nutritional source [13]; however, cherry plums are not so widely diffused due to the low resistance of the fruit in the postharvest management. Given that germplasm conservation is a substantial contribution to preserving knowledge about all crops, the identification of new resources and their characterization is an indispensable tool for achieving sustainable development targets by reducing the risk of genetic erosion. Indigenous genetic diversity is now recognized as a tool for resilience, mitigating the climate crisis due to increased adaptation with less consumption of natural resources, especially soil and water. Genetic diversity can conserve those genes that are potentially useful in strengthening resistance to pathogens or adaptability to stresses [14]. On a global scale, this type of work is also of strategic importance with a view to achieving the Agenda 2030 goals [15]. The conservation of biodiversity of agricultural interest and its morphological and functional characterization is central to SDGs 1, 2, 3, 12, and 14, with the general convergence of all targets toward SDG 13 being related to urgent action on climate change mitigation and adaptation, which, in some ways, underlies all the goals [16].

The attention of consumers toward an increasingly healthy diet has led to an increase in the consumption of fruits, with reference to red fruits not only for their nutritional value but also for their characteristic taste and their well-known health properties [17]. In fact, these fruits, in addition to vitamins and minerals, are rich in compounds with several health properties, mainly phenolic acids such as *p*-coumaric acid, vanillic acid β-glucoside, protocatechuic acid, and caffeic acid, and flavonoids such as catechin, epicatechin, quercetin and cyanidin-3-*O*-glucoside [18,19,20].

Metabolic syndrome (MetS) is a complex disorder that is often associated with insulin resistance, high cholesterol and triglycerides levels, and abdominal obesity [21]. The role of oxidative stress in its pathogenesis was proved [22,23]. Găman et al. [24] evidenced that in the pancreatic β cells of subjects affected by type 2 diabetes, oxidative stress can reduce insulin secretion and, consequently, glucose uptake.

Although research has elucidated many of the mechanisms underlying MetS, its treatment remains a challenge, given the complexity of this disease. For this reason, many research groups are looking for bioactive compounds from food products that can play a preventive role in the onset of this syndrome. Many of these compounds belong to the class of phenols and possess antihypertensive, antihyperglycemic, antihypercholesterolemic, antioxidant, and anti-inflammatory activity, and furthermore, they can produce body weight loss or prevent against body weight gain [25,26].

Among the potentially used preventing approaches to counteract MetS and obesity, the inhibition of α-glucosidase, α-amylase, and lipase was one of the most applied. In fact, the inhibition of carbohydrate hydrolyzing enzymes delays carbohydrate digestion with a consequent hypoglycaemic effect, whereas the inhibition of pancreatic lipase reduces the absorption of ingested fats with a consequent hypolipidemic effect [27,28].

Therefore, herein, we report, for the first time, the pomological characteristics, chemical profile, and nutraceutical properties of different extracts obtained from *Prunus cerasifera* cv ‘Alimena’, a new bloody pulp cultivar from Sicily. The anthocyanin (TAC), flavonoid (TFC), and total phenol (TPC) contents were spectrophotometrically measured. The complete phytochemical profile was assessed using LC-ESI/LTQOrbitrap/MS analysis. A multitarget approach was applied to assess antioxidant activity (ABTS, DPPH, *β*-carotene bleaching, and FRAP assays). The inhibitory activity against key enzymes involved in MetS was also assessed.

## 2. Materials and Methods

### 2.1. Chemicals and Reagents

All chemicals utilized in this study were purchased from VWR International (Milan, Italy) and Sigma-Aldrich Chemical Co., Ltd. (Milan, Italy).

### 2.2. Plant Material

The research was carried out on a new accession of *Prunus cerasifera* L. named ‘Alimena’ and identified in an agricultural area in the territory of Alimena, Sicily (Italy) at an altitude of 675 m s/l, (37°41′25″ N; 14°05′53″ E). From the selected natural tree, budsticks have been collected for developing a small experimental orchard with 50 trees grafted onto *P. cerasifera* myrobalan 29C.

The orchard was established in 2015, and standard growing techniques have been applied since then. The planting density was 5 m × 5 m, fully irrigated during summer and managed by adopting spontaneous cover crops during the winter-spring season. Winter and summer pruning was performed yearly; fruit thinning was not necessarily due to a regular crop density recorded at fruit set.

### 2.3. Morphological Description

Morphological description was carried out by applying the Guidelines for the Characterization of Plant, Livestock and Microbial Genetic Resources approved based on the National Agricultural Biodiversity Plan of the Italian Ministry of Agriculture [29]. The Guidelines were drafted based on the international descriptors approved by UPOV [30] and contain references to plant traits considered essential for the characterization of plum tree accessions with the aim of distinguishing and defining their uniqueness.

Application of the guidelines involves the observation of multiple characters related to the tree and morphological characteristics of vegetative and reproductive organs. Leaves and wood samples were taken from mature trees during the vegetative-reproductive season, and at the same time, all observations of tree habits were recorded. At maturity, a sample of 100 fruits was subjected to morphological analysis (height, width, and thickness of the drupe and stone), as well as to qualitative analysis (skin and pulp color, titratable acidity, and soluble sugar content). For this purpose, fruits were randomly taken from the mass harvested at maturity from 5 plants of the same age. The samples were transported to the laboratory with refrigerated facilities so as not to suffer any damage or spoilage.

### 2.4. Extraction Procedure

A total of 500 g of ripe fruits of *P. cerasifera* were cleaned, stone removed, and homogenized into puree (PA, 380 g) using a food processor. To determine the water quantity, 50 g of puree, once freeze-dried, to give 7.90 g of extract (content of water: 84.2%). Three hundred grams of PA were extracted for seven days at r.t. with 600 mL of acetone. The solvent was evaporated to give, after freeze-drying, 35.5 g of dry extract (PB). Then, 2.5 g of PB, after dilution with deionized water (≈15 mL) and then extracted with butanol (8 × 10 mL). The butanolic extracts were evaporated to give, after freeze-drying, 0.63 g of dry material (PC). The aqueous layer was freeze-dried to give 1.8 g of extract (PD). As an alternative extraction method, 20 g of PA was mixed with organic solution [≈120 mL, acetone/methanol/water/formic acid (40:40:20:0.1, *v*/*v*/*v*/*v*)] [31,32] and the mixture was allowed to stand at 4 °C for 24 h. The crude was filtered and washed with 120 mL of the same solution. The supernatants were combined, evaporated under vacuum at 37 °C, and freeze-dried to obtain 2.51 g of new extract (PF).

### 2.5. Total Phenol (TPC), Flavonoid (TFC), and Anthocyanin Contents (TAC)

Total phenol content (TPC) and total flavonoid content (TFC) were evaluated as previously described by Leporini et al. [33,34]. The differential pH method was used for total anthocyanin content (TAC) quantification [35].

### 2.6. LC-HRMS Analysis

The LC-ESI/HRMS analysis was carried out on a system of liquid chromatography consisting of a Thermo Ultimate RS 3000 UHPLC coupled online to a Q-Exactive hybrid quadrupole Orbitrap high-resolution mass spectrometer (UHPLC-Q-Orbitrap) (Thermo Fisher Scientific, Bremen, Germany), fitted with a HESI II (heated electrospray ionization) probe, working in both negative and positive ionization mode.

In order to allow the chromatographic separation, a Luna C-18 column (RP-18, 2.0 × 150 mm, 5 nm; Waters; Milford, MA, USA), set at a temperature of 30 °C, and a linear gradient, obtained by using mobile phase 0.1% formic acid in water, *v*/*v* (A), and 0.1% formic acid in acetonitrile, *v*/*v* (B), from 5 to 55% of B, in 20 min, at a flow rate of 0.2 mL/min, were used. 5 μL of each extract, dissolved in water/acetonitrile 1:1 *v*/*v* (0.25 mg/mL), were injected by the autosampler.

To allow negative and positive ions analysis, the HESI source parameters were set as follows: spray voltage at −2.50 kV and 3.30 kV, respectively; sheath gas at 50 arbitrary units (a.u.); auxiliary gas at 10 and 15 a.u., respectively; auxiliary gas heater temperature at 300 °C; capillary temperature at 300 °C; S-lens RF value at 50 a.u.

HRMS and HRMS/MS analyses were carried out by experiments of full (mass range: *m*/*z* 150–1400) and data-dependent scan (dd-MS^2^ topN = 5) at resolutions of 70,000 and 17,500, respectively. A normalized collision energy (NCE) of 30 was used. For each sample, three replicates were performed. Data collection and analysis were carried out by using the manufacturer’s software (Xcalibur 2.2).

### 2.7. In Vitro Evaluation of Antioxidant Potential and Relative Antioxidant Capacity Index

The antioxidant activity was assessed by using two radical scavenging test: 2,2-azino-*bis*(3-ethylbenzothiazoline-6-sulfonic acid) (ABTS) and 1,1-diphenyl-2-picrylhydrazyl (DPPH) radical scavenging activity, *β*-carotene bleaching test and Ferric Reducing Ability Power (FRAP). All the procedures were previously detailed [36]. The Relative Antioxidant Capacity Index (RACI) was applied to evaluate the sample characterized by the highest activity [33].

### 2.8. Pancreatic Lipase Inhibitory Activity

The evaluation of pancreatic lipase inhibitory activity was assessed as previously described by Loizzo et al. [37].

### 2.9. Evaluation of α-Amylase and α-Glucosidase Inhibitory Activity

For *α*-amylase and *α*-glucosidase inhibitory activity tests, the procedure previously reported was adopted [36].

### 2.10. Statistical Analysis

Linear regression, assessment of repeatability, calculation of average, relative standard deviation (SD), and Pearson’s correlation coefficient (*r*) were calculated by using Microsoft Excel 2010 software (Redmond, WA, USA). The results were expressed as the means of three different experiments ± SD. The inhibitory concentration 50% (IC_50_) was calculated by using Prism GraphPad Prism version 4.0 for Windows (GraphPad Software, San Diego, CA, USA).

Parametric data were statistically analyzed by using one-way analysis of variance (ANOVA) followed by Tukey’s posthoc test by using Prism GraphPad Prism version 4.0 for Windows. Differences at * *p <* 0.05 were statistically significant, while at ** *p* < 0.01, they were highly significant.

## 3. Results and Discussion

### 3.1. Morphological Data

All data related to the description of morphological traits provided by the applied descriptors are given in Table 1. All data related to the measurements of the fruits, seeds, and leaves, as well as of the fruit quality characteristics, are given in Table 2. The data reported revealed that the ‘Alimena’ accession had morphological characteristics of uniqueness, mainly related to the average fruit size (41.3 g), which is generally larger than that of the other *P. cerasifera* cultivars known in the Mediterranean basin. The color of the skin and pulp, therefore, give these fruits distinguishable traits that are unmatched in the varietal panorama of myrobalans, confirming, moreover, the character of low resistance to handling and transport.

### 3.2. Total Phytochemical Content (TPC, TFC, and TAC) 

The pulp obtained from the *P. cerasifera* ‘Alimena’ fruits was subjected to different extractions with several solvents (acetone, butanol, water, methanol, etc.) to evaluate the impact of the solvent on the phytochemical content. The presence of several compounds with different structures and polarities can drastically affect their solubility [38]. Polar solvents (MeOH, EtOH, and water) were used for the isolation of polyphenols and glycosides from plants. The most common ones are mixtures of water with ethanol and methanol. Ethanol is a good solvent for polyphenol extraction and is safe for human consumption, whereas acetone and ethyl acetate have been used for the extraction of medium molecular weight metabolites, such as terpenoids and flavonoid aglycones [39].

The PF sample resulted in the richest TPC and TFC, with values of 97.63 mg gallic acid equivalent (GAE)/100 g FW and 0.96 mg quercetin equivalent (QE)/100 g FW, respectively, followed by the PC samples, whereas PD was richest in TAC (55.33 mg cyanidine-3-*O*-glucoside/100 g FW) (Table 3).

Previously, the TPC content of *P. cerasifera* ‘Mirabolano’, *P. domestica* cv ‘President’, and *P. salicina* cv ‘Shiro’ at different stages of development was analyzed [40]. All plumes exhibited the highest TPC at the date of commercial harvesting—at about 100 days for ‘Mirabolano’, 130 days for ‘President’, and more than 110 days for “Shiro”. TPC values in a range from 1.34 to 6.11 g/kg FW were found for the red and purple myrobalan plum fruits, respectively [31]. Values from 1.74 to 3.75 g/Kg FW were recorded for the Stanley and French Damson fresh plums, respectively [41].

A lower TPC content was found by Gündüz et al. [8], who investigated *P. cerasifera* selections from Turkey and found values between 136.8 to 583.1 mg GAE/kg FW for ‘Ozark Premier’, and ‘Selection No. 3’, respectively. On the contrary, our data are lower than those found for *P. divaricata* “Demal” and *P. domestica* ‘Sugar plum’, with TPC values of 169.6 and 172.4 mg GAE/100 g, respectively [40]. TFC values from 12.1 to 29.1 mg rutin equivalent/100 g were found for ‘Demal’ and *P. domestica* ‘Red plum’, respectively [42]. TPC values ranging from 177–365 mg GAE/100 g were found for *P. divaricate* yellow and black, respectively [43].

Gil et al. [44] analyzed several Californian flesh plums and found that ‘Black Beaut’ was richer in TPC and TCC in comparison to ‘Angeleno’, ‘Red Beaut’, ‘Wickson’, and ‘Santa Rosa’ cultivars. Moreover, several research papers demonstrated that qualitative and quantitative variability in TPC, is often related to different genetic factors and developmental stages [45].

Our data on TAC are in line with those reported for *P. cerasus* varieties ‘Kántorjánosi’, ‘Újfehértói fürtös’, and ‘Debreceni bötermö’ (TAC values of 21, 56, and 63 mg cyanidine-3-*O*-glucoside/100 g FW, respectively) but lower than those found for the varieties ‘Csengödi csokros’ and ‘Cigánymeggy (TAC values of 295 and 206 mg cyanidine-3-*O*-glucoside/100 g FW, respectively) [46]. A higher TAC was found for *P. domestica* ‘Santa Rosa’ and ‘African Rose’, with values of 164.13 and 326.83 mg cyanidine-3-*O*-glucoside/100 g FW, respectively, and for *P. cerasifera* from Georgia (109.77 mg cyanidine-3-*O*-glucoside/100 g FW) [47,48].

### 3.3. LC-HRMS Analysis

The LC-HRMS profiles of the PC, PD, and PF extracts highlighted the occurrence of several metabolites in *P. cerasifera*, the structures of which could be assigned by a comparison between the molecular formulae, fragmentation patterns, and retention times and the literature data and metabolite databases, allowing us to putatively identify hydroxycinnamic acid derivatives, flavonols, flavan-3-ols and proanthocyanidins, anthocyanins, organic acids, sugar alcohols (and their derivatives), glycosylated hydroxybenzaldehyde and benzylic alcohol derivatives, glycosyl terpenates, and glycosylated aliphatic alcohol derivatives (Table 4) [37,49,50,51,52,53,54]. Except for compounds **28** and **67**, which were previously described in the Rosaceae family [55,56] but not in the genus *Prunus*, and for compounds **35**, **36**, **43**, and **54** described in families other than Rosaceae [57,58,59], to the best of our knowledge, most of these compounds have already been detected in plants belonging to the genus *Prunus* [60,61,62,63,64,65,66,67,68,69,70,71,72,73] but not in the species *cerasifera*. Only compounds **1**, **2**, **5**, **7**, **10**, **12**, **15**, **18**, **20**, **29**, **34**, **42**, **58**, and **75** have already been described in this species [31,45,74,75,76,77,78].

Among the most represented group of metabolites, the derivatives of hydroxycinnamic acid, some differences could be appreciated among the three *P. cerasifera* extracts (Table 4). PF and PC showed, in fact, a higher number of metabolites belonging to this class with respect to the PD extract. It is noteworthy that compounds **21**, **25**, **31**, **35**, and **36**, containing a feruloyl unit in their structure, and compounds **29**, **42**, and **64**, corresponding to methyl ester derivatives of quinic or coumaroyl acid, were not detectable in PD. Moreover, the caffeoyl- and coumaroyl-quinic acid isomers **6**–**7**, and **17**–**19**, along with the caffeoylhexose **8** and the coumaroyl dihexoside isomers **9** and **14** were detectable in PD at a minor intensity level with respect to the other two extracts, as well as the mono-acylated forms of coumaroyldihexoside isomers (**22**, **27**, **32**, and **37**). Furthermore, in PD, the coumaroyldihexoside isomers with two to four acetyl groups were even less intense (**41**, **44**, **47**, **59**, **61**, **65**, **68**–**69**, and **72**–**74**), while the penta-acetylated forms (**76**–**77**) were not detectable at all (Table 4). It is noteworthy that compounds **41**, **44**, and **47** were more evident in PC than in PF.

Regarding the second most representative group of metabolites identified in *P. cerasifera*—flavonols—once again, the PF extract was the most complete of the three, highlighting, besides the quercetin derivatives, the occurrence of isorhamnetin derivatives (**50**, **52**, and **63**) that were not evident in the other two extracts. Furthermore, PC showed the occurrence of flavonols at higher intensity levels than PD (Table 4). Flavan-3-ols (**20** and **34**) and proanthocyanidins A-type (**49**, **56**) occurred in all tested extracts, even if at lower intensity in PD, which lacked the dimers of proanthocyanidins B-type (**11**, **16**, and **23**), which were, in turn, more evident in PF than in PC (Table 4). Glycosylated derivatives of cyanidin and delphinidin (**10**, **12**, **15**, **45**, and **48**) could be observed in PF, PC, and PD, with PC showing the occurrence of the rutinoside derivatives of both anthocyanins (**15** and **45**) at a minor intensity level.

Simple organic acids like **2** and **5** or the nonanedioic acid **70**, as well as derivatives of malic acid with sugar alcohol like sorbitol or galactitol (**3**), or with a ketohexose like fructose (**4**), were detectable in all tested extracts, unlike the glycosylated forms of the dicrotalic and abscisic acids (**54** and **53**), with the first being more evident in PF and PC and the second no detectable in PD (Table 4).

Analogously, the glycosylated derivatives of benzyl alcohol or hydroxybenzaldehyde (**13**, **24**, **26**, and **28**) (differing in sugars) could be highlighted in all the extracts, contrary to glycosylated terpenates (**39** and **46**), which were mainly present in PF and PC, as well as the glycosylated form of hexanol (**43**), likely due to their higher lipophilicity (Table 4).

### 3.4. ‘Alimena’ Myrobalan Antioxidant Potential

The antioxidant activities of myrobalan bloody pulp fruit extracts were assessed using different in vitro methods, namely FRAP, *β*-carotene bleaching test, ABTS, and DPPH assays (Table 5).

The PF sample showed the highest radical scavenging activity, with IC_50_ values of 19.61 and 1.19 μg/mL for the DPPH and ABTS assays, respectively.

In general, by comparing the data obtained from the two different radical scavenging tests, it was possible to note that the DPPH^·^ radical is less sensible than the ABTS^+^ in our samples. In fact, IC_50_ values in the range 19.61–39.02 and 1.19–2.97 μg/mL for the DPPH and ABTS tests, respectively, were obtained. Data from the ABTS test are in the same order of potency as ascorbic acid. The ‘Alimena’ pulp extracts ferric-reducing potential was evaluated by FRAP testing, revealing reduced iron in the samples, with a potency comparable to the positive control BHT.

Pearson’s correlation coefficient evidenced *r* values of 0.79, 0.80, and 0.91 for TPC, FRAP, ABTS, and DPPH, respectively. A similar trend was also observed for TFC, wherein *r* values of 0.73, 0.85, and 0.95 for FRAP, ABTS, and DPPH were recorded, respectively. No positive correlation was found for TAC and the antioxidant data.

Our data on radical scavenging potential are better than those reported for Indian *P. cerasifera* with IC_50_ values of 10.09 and 45.40 μg/mL for ABTS and DPPH assays, respectively [79]. On the contrary, our data obtained by FRAP testing resulted lower than those found in the “Sugar plum” cultivar (563.8 μM Fe (II)/g) [44].

The variability of antioxidant activity during the harvesting of *P. domestica* cv ‘President’, *P. salicina* cv ‘Shiro’, and *P. cerasifera* myrobalan was investigated. By comparing the data on the ‘Alimena’ myrobalan with those obtained by Moscatello et al. [40], it emerges that at the commercial maturity stage, the radical scavenging DPPH activity of our samples is significantly higher, with IC_50_ values in the range 56.22–76.46 µg/mL vs. 19.61–39.02 µg/mL, while similar results can be observed with the plum ‘President’ (IC_50_ in the range 28.63–30.35 µg/mL). Similar results against DPPH were also found for the *P. domestica* ‘African Rose’ and ‘Santa Rosa’ extracts (IC_50_ values of 13.923 and 18.416 μg/mL, respectively) [47]. On the contrary, when comparing this with domesticated *P. domestica,* a higher DPPH activity was observed (IC_50_ in the range 5.2–6.6 µg/mL for black and red fruit, respectively) [43].

Recently, Popović et al. [75] explored different *Prunus* varieties from Serbia and found high variability in the ability of hydroalcoholic extract to counteract the DPPH^·^ radical (IC_50_ in the range 0.83–29.12 mg/mL for steppe cherry and red cherry plum, respectively). However, all data are lower than those found in our extracts.

FRAP values ranging from 11.20 to 44.83 mmol TE/g fresh weight (FW) were found for yellow and purple Chinese myrobalan plum extract, respectively [28], whereas the FRAP values ranged between 0.123 and 0.835 mmol TE/kg FW for ‘Selection 33C 02’ and ‘Selection 31C 18’, respectively [8].

The integration of antioxidant data into the PF sample saw the most active result in terms of antioxidant activity, with the lowest RACI value (0.71) (Figure S1).

### 3.5. Inhibition of Target Enzymes Useful for the Prevention and Treatment of Hyperglycaemia and Obesity

In our continuous search for foods that are able to prevent MeTs, we have investigated the ability of the bloody pulp of myrobalan, a new variety of Sicilian *Prunus*, to counteract the enzymes linked with hyperglycaemia and hyperlipidaemia. All investigated extracts exerted inhibitory enzyme activity in a concentration-dependent manner (Table 6). According to Nowicka et al. [80], the extract richest in flavonols (PF) exerted the highest α-amylase inhibitory activity (IC_50_ value of 34.48 μg/mL). Moreover, the PF sample was found to be more proficient in inhibiting pancreatic lipase, followed by the PD sample, with IC_50_ values of 29.61 and 38.16 μg/mL, respectively. Values from 49.76 to 78.87 μg/mL were found for the PC and PD samples, respectively, against α-glucosidase. The results of correlation analysis evidenced that a strong positive correlation was found between TPC and α-glucosidase inhibitory activity (*r* = 0.96) and TAC and lipase inhibitory property (*r* = 0.80). A weak correlation was found between TFC and α-amylase (*r* = 0.56).

Our data on carbohydrate-hydrolyzing enzymes are in line with those reported by Kołodziejczyk-Czepas [81] for *P. spinosa*; they found IC_50_ values in a range from 15.43 to 90.95 μg/mL for hydroalcoholic extract and butanol fraction, respectively, against α-glucosidase, and from 33.47 to 110.12 μg/mL for ethyl acetate and butanol fraction, respectively, against α-amylase. A lower α-amylase inhibitory effect was found by testing the *P. ceraus* extracts, with IC_50_ values in a range from 330 to 892 μg/mL for the ‘Cigánymeggy’ and ‘Kántorjánosi’ varieties, respectively [46], and by Popović et al. [75], who investigated different *Prunus* species (IC_50_ values in the range 4.61–136.23 mg/mL for *P. fruticose* and *P. pissardi* ‘Carriére’, respectively). In the same work authors tested *Prunus* extracts against *α*-glucosidase and recognized a low inhibitory activity (IC_50_ values in the range 0.41–136.23 mg/mL). 

Podsędek et al. [82] investigated the effect of water extract obtained from *P. persica* fruits from Poland and found lower α-glucosidase inhibitory activity in comparison to our data (IC_50_ value of 264.44 mg/mL) despite a higher TPC content.

Recently, Ullah et al. [83] demonstrated that *P. domestica* subsp. *syriaca* (‘Mirabelle’) was able to inhibit the key enzymes involved in MetS. In particular, the hydroethanolic extract inhibited α-amylase, α-glucosidase, and pancreatic lipase, with IC_50_ values of 7.01, 6.4, 6.0 mg/mL, respectively. All these values are higher than those found in this study.

A perusal analysis of the literature revealed that the inhibition of α-glucosidase should be related to the content of the hydroxycinnamic acid derivatives that are particularly abundant in PF and PC and that exert a more potent inhibitory activity against this enzyme [84].

## 4. Conclusions

The preservation of agro-biodiversity for the purpose of consumption is at the heart of the targets of SDG No. 12 (Responsible Production and Consumption), demonstrating that the spread of a model of sustainability goes through the choices of products that have very high environmental adaptation. For this reason, the identification of new accessions, their description, and in-depth knowledge of their quality characteristics represents a virtuous model of knowledge development for consumption. The growing awareness of consumers toward the possibility of preventing and/or treating pathologies through certain defined foods represents the driving force of the global market for these types of products. In this context, we have analyzed the chemical profile and bioactivity of three extracts of a new bloody pulp selection of myrobalan (*P. cerasifera*). The PF extract showed the most promising bioactivity, which is in agreement with its highest phytochemical content. Further in vivo studies are necessary to better understand the biological properties and potential applications for the development of functional foods or nutraceutical products that are useful to consumers with these types of health problems.

## Figures and Tables

**Table 1:** Application of criteria for describing the morphological traits of plum tree accessions on ‘Alimena’ myrobalan based on a list of descriptors prepared under the National Agricultural Biodiversity Plan.

GlBA Code	UPOV Code	Descriptor	Reference Cultivar	Level of Expression
1	1	Tree vigor		
		High	Valor	7
2	-	Tree habit		
		Upright	Jefferson	2
6	61	Blooming time		
		Early	Ruth Gerstetter	3
10	38	Flower: shape of the petals		
		Rounded	Czar	3
12	17	Leaf blade: Length/Width ratio		
		Medium	D’Ente	5
13	18	Leaf blade: shape		
		Elliptic	D’Ente	2
20	62	Ripening time		
		Early	Bonne de Bry	3
21	43	Fruit dimension		
		Small	Hauszwetsche	3
22	44	Fruit shape		
		Rounded	Fortune	3
23	50	Skin color		
		Red	Victoria	6
24	51	Pulp color		
		Red	Bountiful	6
25	52	Fruit consistency		
		Medium	Zucchella	5
26	53	Pulp sweetness		
		High	Imperial Prune	7
27	54	Seed-pulp adherence		
		Adherent	Jori’s Plum	3
29	56	Seed shape		
		Elliptic large	Regina Claudia	3

All data related to measurements of fruits, seeds, and leaves, as well as for fruit quality characteristics, are given in Table 2.

**Table 2:** Data on morphological descriptive traits related to fruit, seed, and leaf.

	Fruit		Seed		Leaf	
	Length	Width	Length	Width	Length	Width
cm	3.32 ± 0.06	3.41 ± 0.09	2.14 ± 0.02	1.48 ± 0.06	6.11 ± 0.11	3.31 ± 0.04

Data are reported to mean ± Standard Deviation (SD) (*n* = 3).

**Table 3:** Phytochemicals content in the selection of myrobalan bloody pulp fruits extract.

Sample	TPC (mg GAE/100 g FW)	TFC (mg QE/100 g FW)	TAC (Cyanidine-3-*O*-glucoside/100 g FW)
PF	97.63 ± 6.45 ^a^	0.96 ± 0.08 ^a^	39.38 ± 2.09 ^b^
PC	89.61± 2.98 ^b^	0.80 ± 0.06 ^b^	20.24 ± 1.45 ^c^
PD	34.52 ± 2.85 ^c^	0.23 ± 0.04 ^c^	55.33 ± 3.83 ^a^
Sign.	**	**	**

Data are reported as mean ± standard deviation (SD) (*n* = 3). Differences within and between groups were evaluated by one-way ANOVA, followed by Tukey’s multiple-range test. The results followed by different letters in the same column are significantly different. ** Significance at *p* < 0.05.

**Table 4:** Metabolites identified in PF, PC, and PD extracts.

*n*	R_t_ (min)	Compound	Molecular Formula	Error (ppm)	[M − H]^−^ (*m*/*z*)	(−)HRMS/MS	[M + H]^+^ (*m*/*z*)	(+)HRMS/MS	PF	PC	PD
**1**	1.72	Hexose sugar alcohol	C_6_H_14_O_6_	0.34	181.0708	163.0601 (C_6_H_11_O_5_), 101.0231 (C_4_H_5_O_3_), 89.0231 (C_3_H_5_O_3_), 71.0125 (C_3_H_3_O_2_), 59.0126 (C_2_H_3_O_2_)			√√	√√	√√
**2**	1.79	Quinic acid	C_7_H_12_O_6_	0.71	191.0551	173.0445 (C_7_H_9_O_5_), 127.0389 (C_6_H_7_O_3_), 111.0440 (C_6_H_7_O_2_), 93.0333 (C_6_H_5_O), 85.0282 (C_4_H_5_O_2_)			√√	√√	√√
**3**	1.82	(Hexosyl sugar alcohol)-malic acid	C_10_H_18_O_10_	0.32	297.0823	181.0708 (C_6_H_13_O_6_), 133.0131 (C_4_H_5_O_5_), 115.0024 (C_4_H_3_O_4_), 101.0231 (C_4_H_5_O_3_), 89.0231 (C_3_H_5_O_3_), 71.0125 (C_3_H_3_O_2_), 59.0125 (C_2_H_3_O_2_)	321.0793 [M + Na]^+^	205.0687 (C_6_H_14_O_6_Na), 187.0582 (C_6_H_12_O_5_Na), 157.0111 (C_4_H_6_O_5_Na)	√√	√√	√√
**4**	1.85	Ketohexosyl-malic acid	C_10_H_16_O_10_	−0.81	295.0669	179.0553 (C_6_H_11_O_6_), 133.0130 (C_4_H_5_O_5_), 101.0231 (C_4_H_5_O_3_), 89.0231 (C_3_H_5_O_3_), 71.0125 (C_3_H_3_O_2_), 59.0125 (C_2_H_3_O_2_)	319.0633 [M + Na]^+^	301.0535 (C_10_H_14_O_9_Na), 259.0432 (C_8_H_12_O_8_Na), 229.0321 (C_7_H_10_O_7_Na), 203.0530 (C_6_H_12_O_6_Na), 185.0424 (C_6_H_10_O_5_Na), 157.0110 (C_4_H_6_O_5_Na)	√√	√√	√√
**5**	2.61	Citric acid	C_6_H_8_O_7_	2.15	191.0190	111.0075 (C_5_H_3_O_3_), 87.0074 (C_3_H_3_O_3_)			√√	√√	√√
**6**	7.63	3-(*cis*)-*O*-caffeoylquinic acid	C_16_H_18_O_9_	3.87	353.0881	191.0553 (C_7_H_11_O_6_), 179.0340 (C_9_H_7_O_4_), 135.0440 (C_8_H_7_O_2_)			√√	√√	√
**7**	7.94	3-(*trans*)-*O*-Caffeoylquinic acid	C_16_H_18_O_9_	3.86	353.0881	191.0552 (C_7_H_11_O_6_), 179.0341 (C_9_H_7_O_4_), 135.0440 (C_8_H_7_O_2_)			√√	√√	√
**8**	8.43	Caffeoyl hexoside	C_15_H_18_O_9_	3.94	341.0880	179.0339 (C_9_H_7_O_4_), 161.0233 (C_9_H_5_O_3_)			√√	√√	√
**9**	8.65	Coumaroyl dihexoside	C_21_H_28_O_13_	3.82	487.1465	307.0817 (C_15_H_15_O_7_), 163.0399 (C_9_H_7_O_3_), 145.0283 (C_9_H_5_O_2_)			√√	√√	√
**10**	8.74	Cyanidin 3-*O*-hexoside	C_21_H_21_O_11_	1.52	447.0939 [M − 2H]^−^		449.1085 [M]^+^	287.0556 (C_15_H_11_O_6_)	√√	√√	√√
**11**	8.80	Dimeric B-type proanthocyanidins	C_30_H_26_O_12_	3.49	577.1361	289.0721 (C_15_H_13_O_6_)			√√	√	—
**12**	8.98	Cyanidin 3-*O*- hexoside	C_21_H_21_O_11_	−0.24	447.0937 [M − 2H]^−^		449.1077 [M]^+^	287.0553 (C_15_H_11_O_6_)	√√	√√	√√
**13**	9.05	(Benzyl)hexose-hexoside	C_19_H_28_O_11_	2.46	431.1559/477.1615 [(M + FA) − H]^−^	269.1037 (C_13_H_17_O_6_), 161.0445 (C_6_H_9_O_5_), 113.0231 (C_5_H_5_O_3_), 101.0231 (C_4_H_5_O_3_)			√√	√√	√√
**14**	9.10	Coumaroyl dihexoside	C_21_H_28_O_13_	2.57	487.1459	163.0390 (C_9_H_7_O_3_), 145.0283 (C_9_H_5_O_2_)			√√	√√	√
**15**	9.13	Cyanidin 3-*O*-rutinoside	C_27_H_31_O_15_	−0.26	593.1517 [M − 2H]^−^	284.0324 (C_15_H_8_O_6_)	595.1656 [M]^+^	449.1083 (C_21_H_21_O_11_), 287.0551 (C_15_H_11_O_6_)	√√	√	√√
**16**	9.38	dimeric B-type proanthocyanidins	C_30_H_26_O_12_	3.72	577.1362	289.0724 (C_15_H_13_O_6_)			√√	√	—
**17**	9.45	3-*O*-coumaroylquinic acid	C_16_H_18_O_8_	3.16	337.0929	191.0552 (C_7_H_11_O_6_), 163.0390 (C_9_H_7_O_3_), 119.0490 (C_8_H_7_O)			√√	√√	√
**18**	9.72	5-(*trans*)-*O*-caffeoylquinic acid	C_16_H_18_O_9_	3.74	353.0880	191.0553 (C_7_H_11_O_6_)			√√	√√	√
**19**	9.82	4-*O*-caffeoylquinic acid	C_16_H_18_O_9_	3.86	353.0881	191.0553 (C_7_H_11_O_6_), 179.0338 (C_9_H_7_O_4_), 173.0445 (C_7_H_9_O_5_), 135.0440 (C_8_H_7_O_2_)			√√	√√	√
**20**	9.83	Catechin/Epicatechin	C_15_H_14_O_6_	2.89	289.0715	245.0815 (C_14_H_13_O_4_), 205.0500 (C_11_H_9_O_4_), 179.0344 (C_9_H_7_O4)			√√	√√	√
**21**	10.03	5-*O*-Feruloylquinic acid	C_17_H_20_O_9_	3.40	367.1036	193.0499 (C_10_H_9_O_4_), 191.1560 (C_7_H_11_O_6_), 173.0448 (C_7_H_9_O_5_), 161.0231 (C_9_H_5_O_3_), 134.0363 (C_8_H_6_O_2_)			√√	√√	—
**22**	10.19	Coumaroyl-(acetyl)hexose-hexoside isomer	C_23_H_30_O_14_	3.70	529.1571	341.1092 (C_12_H_21_O_11_), 307.0825 (C_15_H_15_O_7_), 163.0389 (C_9_H_7_O_3_), 145.0284 (C_9_H_5_O_2_)			√√	√√	√
**23**	10.35	Dimeric B-type proanthocyanidins	C_30_H_26_O_12_	1.12	577.1347	289.0720 (C_15_H_13_O_6_)			√√	√	—
**24**	10.40	Benzylhexose pentoside	C_18_H_26_O_10_	−1.36	401.1450/447.1504 [(M + FA) − H]^−^	269.1031 (C_13_H_17_O_6_), 161.0446 (C_6_H_9_O_5_)	425.1412 [M + Na]^+^	293.0997 (C_13_H_18_O_6_Na)	√√	√√	√√
**25**	10.48	Feruloyl hexoside isomer	C_16_H_20_O_9_	3.69	355.1037	193.0496 (C_10_H_9_O_4_), 175.0391 (C_10_H_7_O_3_)			√√	√√	—
**26**	10.49	(Benzyl)hexose-dihexoside	C_25_H_38_O_16_	2.00	593.2089	415.1607 (C_19_H_27_O_10_), 269.1032 (C_13_H_17_O_6_)			√√	√√	√√
**27**	10.55	Coumaroyl-(acetyl)hexose-hexoside isomer	C2_3_H_30_O_14_	0.31	529.1570	341.1088 (C_12_H_21_O_11_), 307.0824 (C_15_H_15_O_7_), 163.0391 (C_9_H_7_O_3_), 145.0284 (C_9_H_5_O_2_)	553.1530 [M + Na]^+^	391.1009 (C_17_H_20_O_9_Na), 349.0896 (C_15_H_18_O_8_Na)	√√	√√	√
**28**	10.56	(Benzaldehyde)hexose-pentoside	C_18_H_24_O_11_	3.59	415.1250/461.1310 [(M + FA) − H]^−^	121.0282 (C_7_H_5_O_2_)			√√	√√	√√
**29**	10.59	Methyl caffeoylquinate isomer	C_17_H_20_O_9_	2.92	367.1034	179.0341 (C_9_H_7_O_4_), 173.0449 (C_7_H_9_O_5_), 161.0235 (C_9_H_5_O_3_)			√√	√√	—
**30**	10.65	5-(*cis*)-*O*-caffeoylquinic acid	C_16_H_18_O_9_	3.60	353.0880	191.0553 (C_7_H_11_O_6_)			√√	√√	—
**31**	10.81	Feruloyl hexoside isomer	C_16_H_20_O_9_	3.27	355.1035	193.0502 (C_10_H_9_O_4_), 175.0391 (C_10_H_7_O_3_)			√√	√√	—
**32**	10.85	Coumaroyl-(acetyl)hexose-hexoside isomer	C_23_H_30_O_14_	3.75	529.1572	341.1088 (C_12_H_21_O_11_), 307.0824 (C_15_H_15_O_7_), 163.0391 (C_9_H_7_O_3_), 145.0284 (C_9_H_5_O_2_)			√√	√√	√
**33**	10.90	4-*O*-coumaroylquinic acid	C_16_H_18_O_8_	3.18	337.0929	191.0557 (C_7_H_11_O_6_), 173.0446 (C_7_H_9_O_5_), 163.0387 (C_9_H_7_O_3_)			√√	√√	√√
**34**	10.97	Catechin/Epicatechin	C_15_H_14_O_6_	2.89	289.0715	245.0814 (C_14_H_13_O_4_), 205.0503 (C_11_H_9_O_4_), 179.0344 (C_9_H_7_O_4_)			√√	√√	√
**35**	11.00	Feruloyl-(acetyl)hexose-hexoside isomer	C_24_H_32_O_15_	2.47	559.1671	175.0391 (C_10_H_7_O_3_)			√√	√√	—
**36**	11.26	Feruloyl-(acetyl)hexose-hexoside isomer	C_24_H_32_O_15_	2.85	559.1673	175.0392 (C_10_H_7_O_3_)			√√	√√	—
**37**	11.43	Coumaroyl-(acetyl)hexose-hexoside isomer	C_23_H_30_O_14_	3.75	529.1572	341.1095 (C_12_H_21_O_11_), 307.0820 (C_15_H_15_O_7_), 163.0394 (C_9_H_7_O_3_), 145.0284 (C_9_H_5_O_2_)			√√	√√	√
**38**	11.51	Methyl coumaroylquinate isomer	C_17_H_20_O_8_	3.83	351.1088	145.0283 (C_9_H_5_O_2_)			√√	√√	√√
**39**	11.61	Dimethyl-hydroxy-decadienedioate hexoside	C_18_H_28_O_10_	3.74	403.1614	241.1080 (C_12_H_17_O_5_), 197.1178 (C_11_H_17_O_3_), 181.0866 (C_10_H_13_O_3_), 59.0126 (C_2_H_3_O_2_)			√√	√√	√
**40**	11.81	Methyl coumaroylquinate isomer	C_17_H_20_O_8_	3.83	351.1088	145.0284 (C_9_H_5_O_2_), 119.0492 (C_8_H_7_O)			√√	√√	√√
**41**	11.93	Coumaroyl-(diacetyl)dihexoside isomer	C_25_H_32_O_15_	3.00	571.1675	307.0824 (C_15_H_15_O_7_), 163.0389 (C_9_H_7_O_3_), 145.0284 (C_9_H_5_O_2_)			√√	√√	√
**42**	12.22	Methyl caffeoylquinate isomer	C_17_H_19_O_9_	2.97	367.1034	179.0341 (C_9_H_7_O_4_), 161.0238 (C_9_H_5_O_3_)			√√	√√	—
**43**	12.26	Hexyl-dihexoside isomer	C_18_H_34_O_11_	3.27	471.2084 [(M + FA) − H]^−^	263.1499 (C_12_H_24_O_6_), 161.0444 (C_6_H_9_O_5_), 101.0231 (C_4_H_5_O_3_)			√√	√√	√
**44**	12.37	Coumaroyl-(diacetyl)dihexoside isomer	C_25_H_32_O_15_	2.65	571.1673	341.1088 (C_12_H_21_O_11_), 307.0834 (C_15_H_15_O_7_), 163.0388 (C_9_H_7_O_3_), 145.0283 (C_9_H_5_O_2_)	595.1644 [M + Na]^+^	391.1009 (C_17_H_20_O_9_Na), 349.0900 (C_15_H_18_O_8_Na), 331.0790 (C_15_H_16_O_7_Na), 287.0756 (C_10_H_16_O_8_Na), 167.0315 (C_6_H_8_O_4_Na)	√√	√√	√
**45**	12.50	Delphinidin-3-*O*-rutinoside	C_27_H_31_O_16_	0.18	609.1466 [M − 2H]^−^		611.1608 [M]^+^	303.0501 (C_15_H_11_O_7_)	√√	√	√√
**46**	12.59	Dimethyl-octadienedioate hexoside	C_16_H_24_O_9_	2.93	359.1347	197.0810 (C_10_H_13_O_4_), 153.0910 (C_9_H_13_O_2_), 59.0125 (C_2_H_3_O_2_)			√√	√√	√
**47**	12.64	Coumaroyl-(diacetyl)dihexoside isomer	C_25_H_32_O_15_	1.30	571.1665	341.1084 (C_12_H_21_O_11_), 307.0829 (C_15_H_15_O_7_), 163.0390 (C_9_H_7_O_3_), 145.0284 (C_9_H_5_O_2_)	595.1636 [M + Na]^+^	391.1004 (C_17_H_20_O_9_Na), 349.0899 (C_15_H_18_O_8_Na), 331.0804 (C_15_H_16_O_7_Na), 287.0732 (C_10_H_16_O_8_Na), 167.0317 (C_6_H_8_O_4_Na)	√√	√√	√
**48**	12.66	Delphinidin-3-*O*-hexoside	C_21_H_21_O_12_	0.34			465.1029 [M]^+^	303.0502 (C_15_H_11_O_7_)	√√	√√	√√
**49**	12.79	Dimeric A-type proanthocyanidins	C_30_H_24_O_12_	1.45	575.1204	539.0960 (C_30_H_19_O_10_), 423.0726 (C_22_H_15_O_9_), 407.0771 (C_22_H_15_O_8_), 327.0513 (C_17_H_11_O_7_), 289.0723 (C_15_H_13_O_6_), 287.0562 (C_15_H_11_O_6_), 285.0409 (C_15_H_9_O_6_)	577.1349	287.0552 (C_15_H_11_O_6_)	√√	√√	√
**50**	12.92	Isorhamnetin 3-*O*-rutinoside	C_28_H_32_O_16_	3.78	623.1630	315.0503 (C_16_H_11_O_7_), 314.0431 (C_16_H_10_O_7_)			√√	—	—
**51**	13.08	Quercetin-3-*O*-hexoside	C_21_H_20_O_12_	0.12	463.0889	301.0346 (C_15_H_9_O_7_)	465.1028	303.0502 (C_15_H_11_O_7_)	√√	√√	√
**52**	13.12	Isorhamnetin-3-*O*-pentose-hexoside	C_27_H_30_O_16_	1.18	609.1472	315.0509 (C_16_H_11_O_7_)	611.1614	317.0657 (C_16_H_13_O_7_)	√√	—	—
**53**	13.20	Abscisic acid hexoside	C_21_H_30_O_9_	1.59			449.1789 [M + Na]^+^	287.1258 (C_15_H_20_O_4_Na)	√√	√√	—
**54**	13.26	Dicrotalic acid (Benzyl)hexoside	C_19_H_26_O_10_	3.91	413.1458	269.1039 (C_13_H_17_O_6_)			√√	√√	√
**55**	13.36	Quercetin-3-*O*-di-pentoside	C_25_H_26_O_15_	1.65	565.1207	301.0325 (C_15_ H_9_ O_7_)	567.1354	303.0506 (C_15_ H_11_ O_7+_)	√√	√√	√
**56**	13.43	Dimeric A-type proanthocyanidins	C_30_H_24_O_12_	1.35	575.1204	539.0959 (C_30_H_19_O_10_), 407.0771 (C_22_H_15_O_8_), 327.0507 (C_17_H_11_O_7_), 289.0719 (C_15_H_13_O_6_), 287.0560 (C_15_H_11_O_6_), 285.0405 (C_15_H_9_O_6_)	577.1348	287.0553 (C_15_H_11_O_6_)	√√	√√	√
**57**	13.59	Quercetin-3-*O*-pentoside	C_20_H_18_O_11_	1.98	433.0779	301.0345 (C_15_H_9_O_7_)	435.0930	303.0503 (C_15_H_11_O_7_)	√√	√√	√
**58**	13.68	Quercetin 3-*O*-rutinoside	C_27_H_30_O_16_	1.18	609.1468	301.0349 (C_15_H_9_O_7_)	611.1614	303.0516 (C_15_H_11_O_7_)	√√	√√	√
**59**	13.84	Coumaroyl-(triacetyl)dihexoside isomer	C_27_H_34_O_16_	3.81	613.1786	163.0387 (C_9_H_7_O_3_), 145.0284 (C_9_H_5_O_2_)			√√	√√	√
**60**	14.01	Quercetin-3-*O*-pentoside	C_20_H_18_O_11_	1.06	433.0778	301.0349 (C_15_H_9_O_7_)	435.0927/457.0744 [M + Na]^+^	303.0501 (C_15_H_11_O_7_)	√√	√√	√
**61**	14.16	Coumaroyl-(triacetyl)dihexoside isomer	C_27_H_34_O_16_	1.21	613.1782	383.1196 (C_14_H_23_O_12_), 307.0813 (C_15_H_15_O_7_), 163.0393 (C_9_H_7_O_3_), 145.0284 (C_9_H_5_O_2_)	637.1747 [M + Na]^+^	391.0995 (C_17_H_20_O_9_Na), 373.0882 (C_17_H_18_O_8_Na), 287.0734 (C_10_H_16_O_8_Na), 167.0315 (C_6_H_8_O_4_Na)	√√	√√	√
**62**	14.17	Quercetin-3-*O*-deoxyhexoside	C_21_H_20_O_11_	3.36	447.0937	301.0349 (C_15_H_9_O_7_)	449.1085		√√	√√	√
**63**	14.18	Isorhamnetin 3-*O*-hexoside	C_22_H_22_O_12_	1.60	477.1044	314.0431 (C_16_H_10_O_7_)	479.1192	317.0659 (C_16_H_13_O_7_)	√√	—	—
**64**	14.32	Methyl (hexosyl)-*O*-coumarate isomer	C_16_H_20_O_8_	0.86			363.1054 [M + Na]^+^	201.0524 (C_10_H_10_O_3_Na), 185.0427 (C_6_H_10_O_5_Na)	√√	√√	—
**65**	14.33	Coumaroyl-(triacetyl)dihexoside isomer	C_27_H_34_O_16_	1.03	613.1783	341.1087 (C_12_H_21_O_11_), 163.0390 (C_9_H_7_O_3_), 145.0284 (C_9_H_5_O_2_)	637.1746 [M + Na]^+^	391.0992 (C_17_H_20_O_9_Na), 373.0886 (C_17_H_18_O_8_Na), 287.0739 (C_10_H_16_O_8_Na), 167.0317 (C_6_H_8_O_4_Na)	√√	√√	√
**66**	14.36	Quercetin-3-*O*-(acetyl)hexoside	C_23_H_22_O_13_	1.25	505.0996	300.0273 (C_15_H_8_O_7_)	507.1140	303.0502 (C_15_H_11_O_7_)	√√	√√	√
**67**	14.65	[(Dihydroxybenzoyloxy)-dihydroxyphenyl]acetate methyl ester	C_16_H_14_O_8_	1.54	333.0620	197.0446 (C_9_H_9_O_5_), 165.0183 (C_8_H_5_O_4_)	335.0767	137.0235 (C_7_H_5_O_3_)	√√	√√	√
**68**	14.68	Coumaroyl-(triacetyl)dihexoside isomer	C_27_H_34_O_16_	1.31	613.1783	383.1187 (C_14_H_23_O_12_), 341.1085 (C_12_H_21_O_11_), 163.0390 (C_9_H_7_O_3_), 145.0284 (C_9_H_5_O_2_)	637.1747 [M + Na]^+^	391.0998 (C_17_H_20_O_9_Na), 373.0895 (C_17_H_18_O_8_Na), 287.0758 (C_10_H_16_O_8_Na)	√√	√√	√
**69**	14.91	Coumaroyl-(triacetyl)dihexoside isomer	C_27_H_34_O_16_	1.31	613.1786	383.1197 (C_14_H_23_O_12_), 341.1094 (C_12_H_21_O_11_), 307.0833 (C_15_H_15_O_7_), 163.0391 (C_9_H_7_O_3_), 145.0283 (C_9_H_5_O_2_)	367.1747 [M + Na]^+^	391.1006 (C_17_H_20_O_9_Na), 373.0890 (C_17_H_18_O_8_Na), 287.0744 (C_10_H_16_O_8_Na), 167.0317 (C_6_H_8_O_4_Na)	√√	√√	√
**70**	15.14	Azelaic acid	C_9_H_16_O_4_	2.86	187.0970	125.0960 (C_8_H_13_O)			√√	√√	√√
**71**	15.28	Quercetin-3-*O*-(Acetyl)rutinoside	C_29_H_34_O_17_	1.28	651.1583	301.0351 (C_15_H_9_O_7_)	653.1721		√√	√√	√
**72**	16.62	Coumaroyl-(tetraacetyl)dihexoside isomer	C_29_H_36_O_17_	1.04	655.1893	163.0390 (C_9_H_7_O_3_), 145.0284 (C_9_H_5_O_2_)	679.1852 [M + Na]^+^	373.0889 (C_17_H_18_O_8_Na), 329.0846 (C_12_H_18_O_9_Na)	√√	√√	√
**73**	16.86	Coumaroyl-(tetraacetyl)dihexoside isomer	C_29_H_36_O_17_	1.31	655.1892	163.0395 (C_9_H_7_O3), 145.0285 (C_9_H_5_O_2_)	679.1854 [M + Na]^+^	433.1112 (C_19_H_22_O_10_Na), 373.0940 (C_17_H_18_O_8_Na), 209.0423 (C_8_H_10_O_5_Na)	√√	√√	√
**74**	17.17	Coumaroyl-(tetraacetyl)dihexoside isomer	C_29_H_36_O_17_	0.69	655.1893	383.1197 (C_14_H_23_O_12_), 163.0393 (C_9_H_7_O_3_), 145.0284 (C_9_H_5_O_2_)	679.1849 [M + Na]^+^	433.1115 (C_19_H_22_O_10_Na), 391.1004 (C_17_H_20_O_9_Na), 373.0901 (C_17_H_18_O_8_Na), 329.0835 (C_12_H_18_O_9_Na), 209.0422 (C_8_H_10_O_5_Na)	√√	√√	√
**75**	17.81	Quercetin	C_15_H_10_O_7_	2.73	301.0351	178.9977 (C_8_H_3_O_5_), 151.0025 (C_7_H_3_O_4_), 121.0283 (C_7_H_5_O_2_)			√√	√√	—
**76**	19.76	Coumaroyl-(pentaacetyl)dihexoside isomer	C_31_H_38_O_18_	3.34	697.1998	145.0282 (C_9_H_5_O_2_)			√√	√√	—
**77**	19.95	Coumaroyl-(pentaacetyl)dihexoside isomer	C_31_H_38_O_18_	3.17	697.1996	145.0283 (C_9_H_5_O_2_)			√√	√√	—

Legend: √√ = detectable; √ = detectable at low intensity level (1.1 × 10^3^ < Normalized Level < 4.0 × 10^4^); — = not detectable.

**Table 5:** ‘Alimena’ Myrobalan Antioxidant Potential.

Sample	DPPH Test (IC_50_ μg/mL)	ABTS Test (IC_50_ μg/mL)	*β*-Carotene Bleaching Test (IC_50_ μg/mL)	FRAP Test (μM Fe(II)/g)
PF	19.61 ± 2.98 ^a^	1.19 ± 0.06 ^a^	39.38 ±2.09 ^c^	58.72 ± 2.76 ^b^
PC	29.63 ± 6.45 ^b^	2.46 ± 0.08 ^b^	11.32 ±1.76 ^a^	64.90 ± 3.52 ^c^
PD	39.02 ± 4.45 ^c^	2.97 ± 0.09 ^c^	20.24 ±1.45 ^b^	53.01 ± 2.20 ^a^
Sign.	**	**	**	**
**Positive control**				
Ascorbic acid	5.04 ± 0.81	1.72 ± 0.14		
Propyl gallate			0.09 ± 0.04	
BHT				63.26 ± 2.31

Differences within and between the groups were evaluated by one-way ANOVA followed by Tukey’s multiple-range test. The results followed by different letters in the same column are significantly different. ** Significance at *p* < 0.05.

**Table 6:** Enzymes inhibitory activities by ‘Alimena’ myrobalan extracts.

Sample	Lipase (IC_50_ μg/mL)	α-Amylase (IC_50_ μg/mL)	α-Glucosidase (IC_50_ μg/mL)
PF	29.61 ± 1.45 ^a^	34.48 ± 4.22 ^a^	55.27 ± 2.97 ^b^
PC	78.53 ± 2.96 ^c^	54.49 ± 3.87 ^c^	49.76 ± 2.76 ^a^
PD	38.16 ± 1.89 ^b^	51.50 ± 3.24 ^b^	78.87 ± 3.56 ^c^
Sign.	**	**	**
**Positive control**			
Orlistat	3.74 ± 1.01		
Acarbose		50.12 ± 1.13	35.55 ± 1.10

Data are reported as mean ± standard deviation (SD) (*n* = 3). Differences within and between the groups were evaluated by one-way ANOVA, followed by Tukey’s multiple-range test. The results followed by different letters in the same column are significantly different. ** Significance at *p* < 0.05.

## Data Availability

Data are available from the authors upon reasonable request.

## Supplementary Materials

The following supporting information can be downloaded at: https://www.mdpi.com/article/10.3390/foods12051107/s1, Figure S1. RACI values.

## References

[1] Sottile F., Caltagirone C., Giacalone G., Peano C., Barone E. (2022). Unlocking plum genetic potential: Where are we at?. Horticulturae.

[2] Szymajda M., Studnicki M., Kuras A., Żurawicz E. (2022). Cross-compatibility in interspecific hybridization between three *Prunus* species. S. Afr. J. Bot..

[3] Iacona C., Cirilli M., Zega A., Frioni E., Silvestri C., Muleo R. (2013). A somaclonal myrobalan rootstock increases waterlogging tolerance to peach cultivar in controlled conditions. Sci. Hortic..

[4] Motisi A., Pernice F., Sottile F., Caruso T. (2004). Rootstock effect on stem water potential gradients in cv. “Armking” nectarine trees. Acta Hortic..

[5] Nasri A., Baklouti E., Ben Romdhane A., Maalej M., Schumach H.M., Drira N., Fki L. (2019). Large-scale propagation of Myrobolan *(Prunus cerasifera*) in RITA^®^ bioreactors and ISSR-based assessment of genetic conformity. Sci. Hortic..

[6] Carmona-Martin E., Petri C. (2020). Adventitious regeneration from mature seed-derived tissues of *Prunus cerasifera* and *Prunus insititia*. Sci. Hortic..

[7] Impallari F.M., Monte M., Girgenti V., Del Signore M.B., Sottile F. (2010). Biodiversity of Sicilian fruit trees: Studies on plums. Acta Hortic..

[8] Gündüz K., Saraçoğlu O. (2012). Variation in total phenolic content and antioxidant activity of *Prunus cerasifera* Ehrh. selections from Mediterranean region of Turkey. Sci. Hortic..

[9] Faust M., Suranyi D. (1999). Origin and dissemination of plums. Hortic. Rev..

[10] Horvath A., Balsemin E., Barbot J.C., Christmann H., Manzano G., Reynet P., Laigret F., Mariette S. (2011). Phenotypic variability and genetic structure in plum (*Prunus domestica* L.), cherry plum (*P. cerasifera* Ehrh.) and sloe (*P. spinosa* L.). Sci. Hortic..

[11] Moore G., Tymowski W. (2005). Explanatory Guide to the International Treaty on Plant Genetic Resources for Food and Agriculture.

[12] Gianní S., Sottile F. (2016). In vitro storage of plum germplasm by slow growth. Hort. Sci..

[13] Bandeira Reidel R.V., Cioni P.L., Pistelli L. (2017). Volatile emission of different plant parts and fruit development from Italian cherry plums (*Prunus cerasifera* and *P. cerasifera* ‘Pissardii’). Biochem. System. Ecol..

[14] Priyanka V., Kumar R., Dhaliwal I., Kaushik P. (2021). Germplasm Conservation: Instrumental in Agricultural Biodiversity: A Review. Sustainability.

[15] McCouch S., Navabi Z.K., Abberton M., Anglin N.L., Barbieri R.L., Baum M., Bett K., Booker H., Brown G.L., Bryan G.J. (2020). Mobilizing Crop Biodiversity. Mol. Plant..

[16] Priyadarshan P.M., Jain S.M. (2022). Cash Crops: Genetic Diversity, Erosion, Conservation and Utilization. An Introduction.

[17] Cosme F., Pinto T., Aires A., Morais M.C., Bacelar E., Anjos R., Ferreira-Cardoso J., Oliveira I., Vilela A., Gonçalves B. (2022). Red fruits composition and their health benefits—A review. Foods.

[18] Fu H., Mu X., Wang P., Zhang J., Fu B., Du J. (2020). Fruit quality and antioxidant potential of *Prunus humilis* Bunge accessions. PLoS ONE.

[19] Kayano S., Kikuzaki H., Fukutsuka N., Mitani T., Nakatani N. (2002). Antioxidant activity of prune (*Prunus domestica* L.) constituents and a new synergist. J. Agric. Food Chem..

[20] Varga E., Domokos E., Fogarasi E., Steanesu R., Fülöp I., Croitoru M.D., Laczkó-Zöld E. (2017). Polyphenolic compounds analysis and antioxidant activity in fruits of *Prunus spinosa* L.]. Acta Pharm. Hung..

[21] Jiang X., Yang Z., Wang S., Deng S. (2022). “Big Data” approaches for prevention of the metabolic syndrome. Front. Genet..

[22] Scuteri A., Laurent S., Cucca F., Cockcroft J., Cunha P.G., Mañas L.R., Mattace Raso F.U., Muiesan M.L., Ryliškytė L., Rietzschel E. (2015). Metabolic Syndrome and arteries research (MARE) consortium. Metabolic syndrome across Europe: Different clusters of risk factors. Eur. J. Prev. Cardiol..

[23] Carrier A. (2017). Metabolic syndrome and oxidative stress: A complex relationship. Antioxid. Redox Signal..

[24] Găman M., Epingeac M., Diaconu C., Găman A. (2019). Oxidative stress levels are increased in type 2 diabetes mellitus and obesity. J. Hypertens.

[25] Del Rio D., Rodriguez-Mateos A., Spencer J.P., Tognolini M., Borges G., Crozier A. (2013). Dietary (poly)phenolics in human health: Structures, bioavailability, and evidence of protective effects against chronic diseases. Antioxid. Redox Signal..

[26] Rahman M.M., Rahaman M.S., Islam M.R., Rahman F., Mithi F.M., Alqahtani T., Almikhlafi M.A., Alghamdi S.Q., Alruwaili A.S., Hossain M.S. (2021). Role of phenolic compounds in human disease: Current knowledge and future prospects. Molecules.

[27] Tundis R., Loizzo M.R., Menichini F. (2010). Natural products as alpha-amylase and alpha- glucosidase inhibitors and their hypoglycaemic potential in the treatment of diabetes: An update. Mini-Rev. Med. Chem..

[28] de la Garza A.L., Milagro F.I., Boque N., Campión J., Martínez J.A. (2011). Natural inhibitors of pancreatic lipase as new players in obesity treatment. Planta Med..

[29] RRN https://www.reterurale.it/flex/cm/pages/ServeBLOB.php/L/IT/IDPagina/9580.

[30] UPOV https://www.upov.int/edocs/tgdocs/en/tg041.pdf.

[31] Wang Y., Chen X., Zhang Y., Chen X. (2012). Antioxidant activities and major anthocyanins of myrobalan plum (*Prunus cerasifera* Ehrh.). J. Food Sci..

[32] Koca I., Karadeniz B. (2009). Antioxidant properties of blackberry and blueberry fruits grown in the Black Sea Region of Turkey. Sci. Hortic..

[33] Leporini M., Loizzo M.R., Sicari V., Pellicanò T.M., Reitano A., Dugay A., Deguin B., Tundis R. (2020). *Citrus clementina* Hort. Juice Enriched with its by-products (peels and leaves): Chemical composition, in vitro bioactivity, and impact of processing. Antioxidants.

[34] Leporini M., Tundis R., Sicari V., Pellicanò T.M., Dugay A., Deguin B., Loizzo M.R. (2020). Impact of extraction processes on phytochemicals content and biological activity of *Citrus clementina* Hort. Ex Tan. leaves: New opportunity for under-utilized food by-products. Food Res. Int..

[35] Loizzo M.R., Sicari V., Pellicanò T., Xiao J., Poiana M., Tundis R. (2019). Comparative analysis of chemical composition, antioxidant and anti-proliferative activities of Italian *Vitis vinifera* by-products for a sustainable agro-industry. Food Chem. Toxicol..

[36] Formoso P., Tundis R., Pellegrino M., Leporini M., Sicari V., Romeo R., Gervasi L., Corrente G., Beneduci A., Loizzo M.R. (2022). Preparation, characterization, and bioactivity of *Zingiber officinale* Roscoe (white ginger) powder based Pickering emulsions. J. Sci. Food Agr..

[37] Loizzo M.R., Tundis R., Leporini M., D’Urso G., Gagliano Candela R., Falco T., Piacente S., Bruno M., Sottile F. (2021). Almond (*Prunus dulcis* cv. *Casteltermini*) skin confectionery by-products: New opportunity for the development of a functional blackberry (*Rubus ulmifolius* Schott) jam. Antioxidants.

[38] Turkmen N., Sari F., Velioglu Y.S. (2006). Effects of extraction solvents on concentration and antioxidant activity of black and black mate tea polyphenols determined by ferrous tartrate and FolineCiocalteu methods. Food Chem..

[39] Dai J., Mumper R.J. (2010). Plant phenolics: Extraction, analysis and their antioxidant and anticancer properties. Molecules.

[40] Moscatello S., Frioni T., Blasi F., Proietti S., Pollini L., Verducci G., Rosati A., Walker R.P., Battistelli A., Cossignani L. (2019). Changes in absolute contents of compounds affecting the taste and nutritional properties of the flesh of three plum species throughout development. Foods.

[41] Kim D.O., Jeong S.W., Lee C.Y. (2003). Antioxidant capacity of phenolic phytochemicals from various cultivars of plums. Food Chem..

[42] Murathan Z.A., Mehmet E.N. (2020). Analyzing biological properties of some plum genotypes grown in turkey. Int. J. Fruit Sci..

[43] Smanalieva J., Iskakova J., Oskonbaeva Z., Wichern F., Darr D. (2019). Determination of physicochemical parameters, phenolic content, and antioxidant capacity of wild cherry plum (*Prunus divaricata* Ledeb.) from the walnut-fruit forests of Kyrgyzstan. Eur. Food Res. Technol..

[44] Gil M.I., Toma Barberan F.A., Hess-Pierce B., Kaderm A.A. (2012). Antioxidant capacities, phenolic compounds, carotenoids, and vitamin C contents of nectarine, peach, and plum cultivars from California. J. Agric. Food Chem..

[45] Celik F., Gundogdu M., Alp Ş., Muradoğlu F., Geçer M., Canan I. (2017). Determination of phenolic compounds, antioxidant capacity and organic acids contents of *Prunus domestica* L., *Prunus cerasifera* Ehrh. and *Prunus spinosa* L. fruits by HPLC. Acta Chromatogr..

[46] Homoki J.R., Nemes A., Fazekas E., Gyémánt G., Balogh P., Gál F., Al-Asri J., Mortier J., Wolber G., Babinszky L. (2016). Anthocyanin composition, antioxidant efficiency, and α-amylase inhibitor activity of different Hungarian sour cherry varieties (*Prunus cerasus* L.). Food Chem..

[47] El-Beltagi H., El- Ansary A., Mostafa M., Kamel T., Safwat G. (2018). Evaluation of the Phytochemical, Antioxidant, Antibacterial and Anticancer Activity of Prunus domestica Fruit. Not. Bot. Horti. Agrobot. Cluj Napoca.

[48] Putkaradze J., Diasamidze M., Vanidze M., Kalandia A. (2021). Antioxidant Activity of *Prunus cerasifera* products. Int. J. Life Sci..

[49] Napolitano A., Di Napoli M., Castagliuolo G., Badalamenti N., Cicio A., Bruno M., Piacente S., Maresca V., Cianciullo P., Capasso L. (2022). The chemical composition of the aerial parts of *Stachys spreitzenhoferi* (Lamiaceae) growing in Kythira Island (Greece), and their antioxidant, antimicrobial, and antiproliferative properties. Phytochemistry.

[50] Navarro-Hoyos M., Arnáez-Serrano E., Quesada-Mora S., Azofeifa-Cordero G., Wilhelm-Romero K., Quirós-Fallas M.I., Alvarado-Corella D., Vargas-Huertas F., Sánchez-Kopper A. (2021). Polyphenolic QTOF-ESI MS characterization and the antioxidant and cytotoxic activities of *Prunus domestica* commercial cultivars from Costa Rica. Molecules.

[51] D’Urso G., Napolitano A., Cannavacciuolo C., Masullo M., Piacente S. (2020). Okra fruit: LC-ESI/LTQOrbitrap/MS/MSn based deep insight on polar lipids and specialized metabolites with evaluation of anti-oxidant and antihyperglycemic activity. Food Funct..

[52] Cerulli A., Napolitano A., Hosek J., Masullo M., Pizza C., Piacente S. (2021). Antioxidant and in vitro preliminary anti-inflammatory activity of *Castanea sativa* (Italian Cultivar “Marrone di Roccadaspide” PGI) burs, leaves, and chestnuts extracts and their metabolite profiles by LC-ESI/LTQOrbitrap/MS/MS. Antioxidants.

[53] Sun J., Lin L., Chen P. (2012). Study of the mass spectrometric behaviors of anthocyanins in negative ionization mode and its applications for characterization of anthocyanins and non-anthocyanin polyphenols. Rapid Commun. Mass Spectrom..

[54] Gómez-Caravaca A.M., Verardo V., Segura-Carretero A., Caboni M.F., Fernández-Gutiérrez A. (2008). Development of a rapid method to determine phenolic and other polar compounds in walnut by capillary electrophoresis–electrospray ionization time-of-flight mass spectrometry. J. Chromatogr. A.

[55] Bijttebier S., Van der Auwera A., Voorspoels S., Noten B., Hermans N., Pieters L., Apers S. (2016). A first step in the quest for the active constituents in *Filipendula ulmaria* (Meadowsweet): Comprehensive phytochemical identification by liquid chromatography coupled to quadrupole-orbitrap mass spectrometry. Planta Med..

[56] Shi Q.Q., Lu S.Y., Peng X.R., Zhou L., Qiu M.H. (2021). Hydroxynitrile glucosides: Bioactive constituent recovery from the oil residue of *Prinsepia utilis*. J. Agric. Food Chem..

[57] Cho J.G., Cha B.J., Seo W.D., Jeong R.H., Shrestha S., Kim J.Y., Kang H.C., Baek N.I. (2015). Feruloyl sucrose esters from *Oryza sativa* roots and their tyrosinase inhibition activity. Chem. Nat. Compd..

[58] Tao S., Huang Y., Chen Z., Chen Y., Wang Y., Wang Y. (2015). Rapid identification of anti-inflammatory compounds from Tongmai Yangxin Pills by liquid chromatography with high-resolution mass spectrometry and chemometric analysis. J. Sep. Sci..

[59] Wu X., Wang Y., Huang X.J., Fan C.L., Wang G.C., Zhang X.Q., Zhang Q.W., Ye W.C. (2011). Three new glycosides from *Hylocereus undatus*. J. Asian Nat. Prod. Res..

[60] Zhang X., Su M., Du J., Zhou H., Li X., Zhang M., Hu Y., Ye Z. (2023). Profiling of naturally occurring proanthocyanidins and other phenolic compounds in a diverse peach germplasm by LC-MS/MS. Food Chem..

[61] Ortega-Vidal J., Cobo A., Ortega-Morente E., Gálvez A., Martínez-Bailén M., Salido S., Altarejos J. (2022). Antimicrobial activity of phenolics isolated from the pruning wood residue of European plum (*Prunus domestica* L.). Ind. Crops Prod..

[62] De Leo M., Iannuzzi A.M., Germano M.P., D’Angelo V., Camangi F., Sevi F., Diretto G., De Tommasi N., Braca A. (2021). Comparative chemical analysis of six ancient italian sweet cherry (*Prunus avium* L.) varieties showing antiangiogenic activity. Food Chem..

[63] Wojdyło A., Nowicka P., Turkiewicz I.P., Tkacz K. (2021). Profiling of polyphenols by LC-QTOF/ESI-MS, characteristics of nutritional compounds and in vitro effect on pancreatic lipase, α-glucosidase, α-amylase, cholinesterase and cyclooxygenase activities of sweet (*Prunus avium*) and sour (*P. cerasus*) cherries leaves and fruits. Ind. Crops Prod..

[64] Tiboni M., Coppari S., Casettari L., Guescini M., Colomba M., Fraternale D.E., Gorassini A., Verardo G., Ramakrishna S., Guidi L. (2021). *Prunus spinosa* extract loaded in biomimetic nanoparticles evokes in vitro anti-inflammatory and wound healing activities. Nanomaterials.

[65] Bottone A., Montoro P., Masullo M., Pizza C., Piacente S. (2020). Metabolite profiling and antioxidant activity of the polar fraction of Italian almonds (Toritto and Avola): Analysis of seeds, skins, and blanching water. J. Pharm. Biomed. Anal..

[66] Blackhall M.L., Berry R., Davies N.W., Walls J.T. (2018). Optimized extraction of anthocyanins from reid fruits’ *Prunus avium* ‘Lapins’ cherries. Food Chem..

[67] Crupi P., Bleve G., Tufariello M., Corbo F., Clodoveo M.L., Tarricone L. (2018). Comprehensive identification and quantification of chlorogenic acids in sweet cherry by tandem mass spectrometry techniques. J. Food Comp. Anal..

[68] Zhang X., Lin Z., Fang J., Liu M., Niu Y., Chen S., Wang H. (2015). An on-line high-performance liquid chromatography–diode-array detector–electrospray ionization–ion-trap–time-of-flight–mass spectrometry–total antioxidant capacity detection system applying two antioxidant methods for activity evaluation of the edible flowers from *Prunus mume*. J. Chromatogr. A.

[69] Kayano S., Kikuzaki H., Hashimoto S., Kasamatsu K., Ikami T., Nakatani N. (2014). Glucosyl terpenates from the dried fruits of *Prunus domestica* L.. Phytochem. Lett..

[70] Treutter D., Wang D., Farag M.A., Argueta Baires G.D., Rühmann S., Neumüller M. (2012). Diversity of phenolic profiles in the fruit skin of *Prunus domestica* plums and related species. J. Agric. Food Chem..

[71] Olszewska M., Kwapisz A. (2011). Metabolite profiling and antioxidant activity of *Prunus padus* L. flowers and leaves. Nat. Prod. Res..

[72] Kikuzaki H., Kayano S., Fukutsuka N., Aoki A., Kasamatsu K., Yamasaki Y., Mitani T., Nakatani N. (2004). Abscisic acid related compounds and lignans in prunes (*Prunus domestica* L.) and their oxygen radical absorbance capacity (ORAC). J. Agric. Food Chem..

[73] Purohit M.C., Rawat M.S.M., Pant G., Nautiyal A.K., Sakakibaraa J., Kaiya T. (1993). A methyl ester of melilotoside from the sapwood of *Prunus cornuta*. Phytochemistry.

[74] Lo Piccolo E., Araniti F., Landi M., Massai R., Guidi L., Abenavoli M.R., Remorini D. (2021). Girdling stimulates anthocyanin accumulation and promotes sugar, organic acid, amino acid level and antioxidant activity in red plum: An overview of skin and pulp metabolomics. Sci. Hortic..

[75] Popović B.M., Blagojević B., Kucharska A.Z., Agić D., Magazin N., Milović M., Serra A.T. (2021). Exploring fruits from genus *Prunus* as a source of potential pharmaceutical agents—In vitro and in silico study. Food Chem..

[76] Shen J., Zhang P., Zhang X., Yao J., Chang J.-M. (2021). Extraction process and composition identification of anthocyanins from Xinjiang wild *Prunus cerasifera* fruit peel. Pak. J. Pharm. Sci..

[77] Liu W., Nisar M.F., Wan C. (2020). Characterization of phenolic constituents from *Prunus cerasifera* Ldb. leaves. J. Chem..

[78] Chen F.F., Sang J., Zhang Y., Sang J. (2018). Development of a green two-dimensional HPLC-DAD/ESI-MS method for the determination of anthocyanins from *Prunus cerasifera* var. *atropurpurea* leaf and improvement of their stability in energy drinks. Int. J. Food Sci. Technol..

[79] Saraswathi K., Sivaraj C., Arumugam P. (2020). Antioxidant and antibacterial activities of ethanol fruit extract of cherry Plum—*Prunus cerasifera* Ehrh. J. Drug Deliv. Ther..

[80] Nowicka P., Wojdyło A., Samoticha J. (2016). Evaluation of phytochemicals, antioxidant capacity, and antidiabetic activity of novel smoothies from selected *Prunus* fruits. J. Funct. Foods.

[81] Kołodziejczyk-Czepas J., Skrobacz K., Czerwińska M.E., Rutkowska M., Prokop A., Michel P., Olszewska M.A. (2022). Valorisation of the Inhibitory Potential of Fresh and Dried Fruit Extracts of *Prunus spinosa* L. towards Carbohydrate Hydrolysing Enzymes, Protein Glycation, Multiple Oxidants and Oxidative Stress-Induced Changes in Human Plasma Constituents. Pharmaceuticals.

[82] Podsędek A., Majewska I., Redzynia M., Sosnowska D., Koziołkiewicz M. (2014). In Vitro Inhibitory Effect on Digestive Enzymes and Antioxidant Potential of Commonly Consumed Fruits. J. Agric. Food Chem..

[83] Ullah H., Sommella E., Santarcangelo C., D’Avino D., Rossi A., Dacrema M., Minno A.D., Di Matteo G., Mannina L., Campiglia P. (2022). Hydroethanolic extract of *Prunus domestica* L.: Metabolite profiling and in vitro modulation of molecular mechanisms associated to cardiometabolic diseases. Nutrients.

[84] Wang T., Li X., Zhou B., Li H., Zeng J., Gao W. (2015). Anti-diabetic activity in type 2 diabetic mice and α-glucosidase inhibitory, antioxidant and anti-inflammatory potential of chemically profiled pear peel and pulp extracts (*Pyrus* spp.). J. Funct. Foods.

