# Supplementary figures and images for: A New Bloody Pulp Selection of Myrobalan (Prunus cerasifera L.): Pomological Traits, Chemical Composition, and Nutraceutical Properties
Source: Foods. 2023 Mar 5;12(5):1107. doi: 10.3390/foods12051107 (PMC10001106; doi:10.3390/foods12051107)

Supplementary

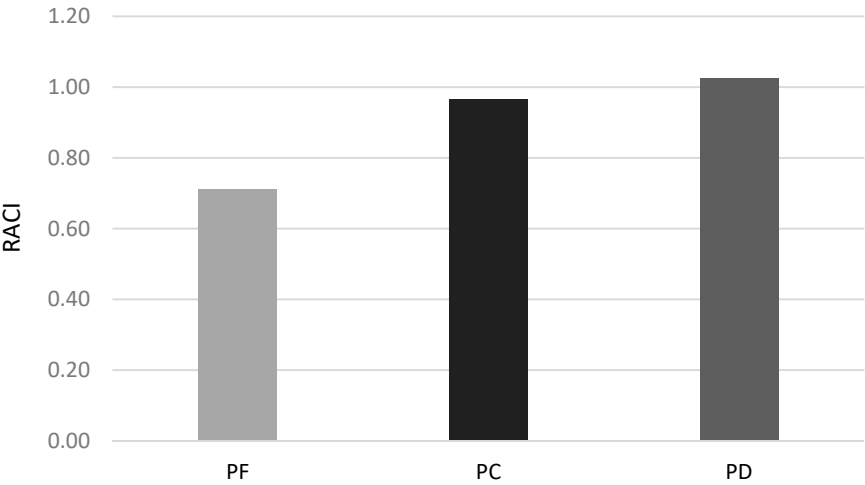

Figure S1. RACI

Supplement: Supplementary file 1 [file foods-12-01107-s001.zip › foods-2244152-supplementary.pdf]
